# Nanoencapsulation and Nanocoating of Bioactives of Application Interest in Food, Nutraceuticals and Pharma

**DOI:** 10.3390/nano14030313

**Published:** 2024-02-04

**Authors:** Cristina Prieto, Jose M. Lagaron

**Affiliations:** Novel Materials and Nanotechnology Group, Institute of Agrochemistry and Food Technology (IATA), Spanish Council for Scientific Research (CSIC), Calle Catedrático Agustín Escardino Benlloch 7, 46980 Paterna, Spain

Bioactives are functional molecules that pose several challenges, including poor solubility, low permeability, and low chemical, biochemical, or process stability, resulting in reduced functionality and bioavailability [1]. In this context, nanoencapsulation and nanocoating technologies emerge as innovative approaches aimed at protecting, processing, masking undesirable properties, controlling release and phase morphology, and, overall, enhancing the bioavailability and functionality of these molecules in applications relevant to the food, nutraceuticals, and pharmaceutical sectors.

Among the bioactive compounds dealt with in this Special Issue, one of the most important and challenging functional ingredients, especially in food and nutraceutical applications, are omega-3 rich oils due to their well-documented health benefits and their susceptibility to oxidation. Consequently, their protection through nanoencapsulation or nanocoating constitutes a very interesting alternative. In this line, Escobar-Garcia et al. [2] proposed the nanoencapsulation of eicosapentaenoic acid (EPA)-rich oil into whey protein concentrate (WPC) by means of emulsion electrospraying assisted by pressurized gas (EAPG). This technology is a simple, continuous, and versatile process that combines electrospraying with gas-driven nebulization. It is performed at ambient temperature, which prevents bioactivity losses, provides free-flowing powder, and has recently become available at an industrial scale. By means of this technology, 80% of EPA-rich oil was encapsulated into WPC, without suffering from oxidation, as demonstrated by the peroxide value of the oil. Due to this encapsulation into WPC, the oxidative and thermal stability of the bioactive compound increased, and the encapsulated product showed a reduced organoleptic impact through the use of rehydrated powdered milk as a model food product. Additionally, the organoleptic impact of mixtures of EPA-rich oil microcapsules and docosahexaenoic acid (DHA)-rich oil microcapsules were tested, and the results showed a global reduced organoleptic impact compared to pure EPA or mixtures of pure EPA and pure DHA. Consequently, the authors highlighted the potential of these encapsulates for their use in personalized medicines or nutraceuticals. Following this research line, Prieto et al. [3] studied another alternative to protect and stabilize omega-3 oils. To be more precise, they studied the effect of the protein purity of whey protein on the characteristics of DHA-rich algae oil encapsulates obtained via the same technology: the room-temperature EAPG. The authors evaluated the effect of three different commercial grades of whey protein purity—35, 80, and 90 wt.%—on the morphology, porosity, encapsulation efficiency, and oxidation and thermal stability. According to the results, protein purity had an important effect on the sphericity, surface smoothness, and porosity. The authors observed that as the protein purity increased, the sphericity and surface smoothness of the particles increased, while porosity decreased. Additionally, protein purities over 80% also provided increased oxidation stability—results that were consistent with the porosity and oxygen permeation results. Moreover, high protein purities showed an increased thermal stability. These results confirmed the importance of the adequate selection of the wall material together with the encapsulation method, and the authors recommended protein contents over 80% for optimal oil stability.

Other examples of highly sensitive bioactive compounds are therapeutic proteins and peptides, which are of great clinical importance due to their crucial role in multiple physiological processes as they are also related to a broad range of pathological conditions that are caused by protein dysfunction or by their presence at non-optimal concentrations. Thus, when developing functional products containing these bioactive compounds, it is important to protect them to maintain their functional activity. In this sense, Schlosser et al. [4] proposed the development of solid formulations by means of electrospraying to stabilize enzymes at different storage temperatures. The authors indicated that this encapsulation technique could be more appropriate for enzymes in comparison with lyophilization and spray drying, since the process is performed at ambient temperature, avoiding heat-induced protein degradation; furthermore, the low flow rate and low atomization stress do not affect the protein structure, and generally result in high encapsulation efficiencies. Therefore, their aim was to evaluate the effect of the electrosprayed solution formulation using a well-known enzyme, i.e., a catalase from bovine liver. Among the components of the solution, the authors analyzed the effect of the type and concentration of surfactant, solvent, encapsulating polymer, and the presence of trehalose on the residual protein content and its residual activity. The authors found that the composition of the solution highly affected the bioactivity, whereby the effect of the type of polymer was particularly significant. Polyvinylpirrolidone encapsulates provided the best results in maintaining enzyme activity, and the addition of trehalose increased the storage stability. Another approach was presented by Ryong Kang et al. [5], who proposed coating liposomes encapsulating a lipopeptide with chitosan to improve the encapsulation efficiency. The liposomes loaded with the lipopeptide obtained from *Bacillus subtilis* KB21 were coated with chitosan through self-assembling. The application of the coating allowed the encapsulates to increase in stability, encapsulation efficiency, and to obtain a slower release profile.

Nevertheless, nanoencapsulation and nanocoating not only serve to protect bioactive compounds, but also to enhance bioavailability, provide a controlled delivery to a targeted section, and improve transport through natural barriers. This is very interesting, since a large proportion of the novel drug candidates present low water solubility or a strong first-pass effect, which reduce their bioavailability [6]. In this regard, Tikhonova et al. [7] studied the nanoencapsulation of indomethacin into phospholipid nanoparticles by means of lyophilization. The encapsulation of this drug will help to avoid side effects, such as cardiovascular and gastrointestinal disorders, as well as enhance bioavailability, consequently increasing bioactivity. In their work, the authors evaluated the morphological characteristics and the physicochemical properties of the nanoparticles The authors managed to produce nanoparticles with an average size of 22 nm without affecting the physicochemical parameters of the drug and the phospholipid. The obtained nanoparticles resulted in a vesicular system, which was stable, with changes in concentration and temperature, as characterized by small-angle X-ray scattering (SAXS). A second proposal to enhance bioavailability was provided by Pardo-Figuerez et al. [8], who proposed the development of an electrospun patch to deliver active pharmaceutical ingredients (APIs) into the oral mucosa, which is an emerging alternative administration route to avoid the first-pass effect from which some drugs suffer. The selected technology is a high-throughput process to produce electrospun fibers to generate a mucoadhesive system that allows for the encapsulation of drugs with high loading and cost-effectiveness. In this work, a three-layer patch composed by an API layer, a mucoadhesive layer, and a backing layer was developed. Carvedilol was selected as a model drug, and non-water-soluble polymers (polycaprolactone, polyhydroxybutyrate, and poly(DL-lactide)) were tested for the reservoir layer in order to provide an adequate release profile. The combination of biopolymers with the amorphization of the drug provided an enhanced release kinetics and unique mucoadhesive properties that made this novel formulation an interesting vehicle for drugs which, among other challenges, suffer from a strong first-pass effect.

In addition, lipid nanometric systems have demonstrated that they can be used as an adjuvant for cosmetic or pharmaceutical purposes in topical applications. In the review prepared by Souto et al. [9], the advantages of the combination of bioactive compounds with lipid nanometric systems such as liposomes, solid lipid nanoparticles (SLNs), and nanostructured lipid carriers (NLCs) in photoprotection and skin anti-aging are presented. The authors indicate that the encapsulation of highly well-known cosmetic bioactive compounds into liposomes could serve as a possible strategy to enhance in vivo antioxidant and anti-aging effects. On the other hand, the combination of physical or chemical sunscreens with SLNs or their encapsulation in these particular systems resulted in synergistic effects, probably due to their composition and their nanometric size range. Additionally, NLCs have shown high hydration capacities and are effective in wrinkle prevention when encapsulating bioactive molecules for cosmetic purposes. However, more studies are required to improve the existing formulations.

Finally, another important factor in preserving bioactive properties is the packaging of the developed functional products. For this reason, an increased number of innovations are focused on the development of packaging with enhanced functionalities that may help increase quality, safety, and extend the product’s shelf-life. Hence, Figueroa-López et al. [10] developed novel and organic recyclable active food packaging films based on electrospun nanofibers of poly(hydroxybutyrate-co-3-hydroxyvalerate) by incorporating cerium oxide nanoparticles. These nanoparticles acted as oxygen scavengers due to their ability to transition from Ce^3+^ to Ce^4+^ states reversibly, which is responsible for their active functional properties. The authors explored the capacities of the developed biopolymeric composites in terms of oxygen-scavenging properties, as well as antioxidant, antimicrobial, barrier, thermal, and mechanical properties. Their findings revealed that the developed materials exhibited antimicrobial and antioxidant properties, an improved water barrier, and an oxygen-scavenging capacity, thus making them excellent candidates to fulfill the aforementioned purposes. Another proposal was provided by Lafraya et al. [11], who developed food-contact super-repellent biopolymeric films with enhanced barrier properties. The authors evaluated three biopolymers, i.e., polylactide, polycaprolactone, and poly(3-hydroxybutyrate-co-3-hydroxyvalerate), combined with hydrophobic silica microparticles to produce this novel material by means of a combination of electrospinning and electrospraying. Superhydrophobic properties with contact angles higher than 155° and sliding angles lower than 10° were achieved for all formulations. Poly(3-hydroxybutyrate-co-3-hydroxyvalerate) presented the best performance in terms of water vapor permeance, resistance to peeling test, and food super-repelling properties to water, yogurt, and custard. The developed material constituted a successful alternative for packaging, which may not only contribute to preserving the food’s organoleptic, nutritious, and healthy properties, but may also contribute to reducing waste and ease recyclability. The last approach in this research line was conducted by Vázquez-González et al. [12], who proposed the use of a bacterial exopolysaccharide, i.e., FucoPol, for food, agriculture, pharmaceutical, or cosmetic purposes. FucoPol is produced by *Enterobacter* A47 and has interesting functional properties, such as emulsion, film-forming, thickening, and flocculating, along with biological activity and adhesive and photoprotective properties. For this reason, the authors studied the electrospinnability of this polysaccharide alone or in combination with other polymers, such as polyethylene oxide and pullulan. FucoPol was found to be an amorphous biopolymer that is stable until 220°, but unable to produce electrospun fibers alone due to its low water solubility and lack of molecular entanglements. However, blends with the proposed biopolymers produced non-agglomerated and homogeneous nanofibers, which could be interesting for the development of novel materials with functional properties.

In conclusion, this Special Issue entitled “Nanoencapsulation and Nanocoating of Bioactives of Application Interest in Food, Nutraceuticals, and Pharma” highlights the transformative potential of nanotechnology in redefining current bioactive formulations. It is anticipated that this compilation will inspire new interdisciplinary research endeavors focused on harnessing nanotechnology applied to bioactive molecules for the advancement of food, nutraceutical, or pharmaceutical products.
